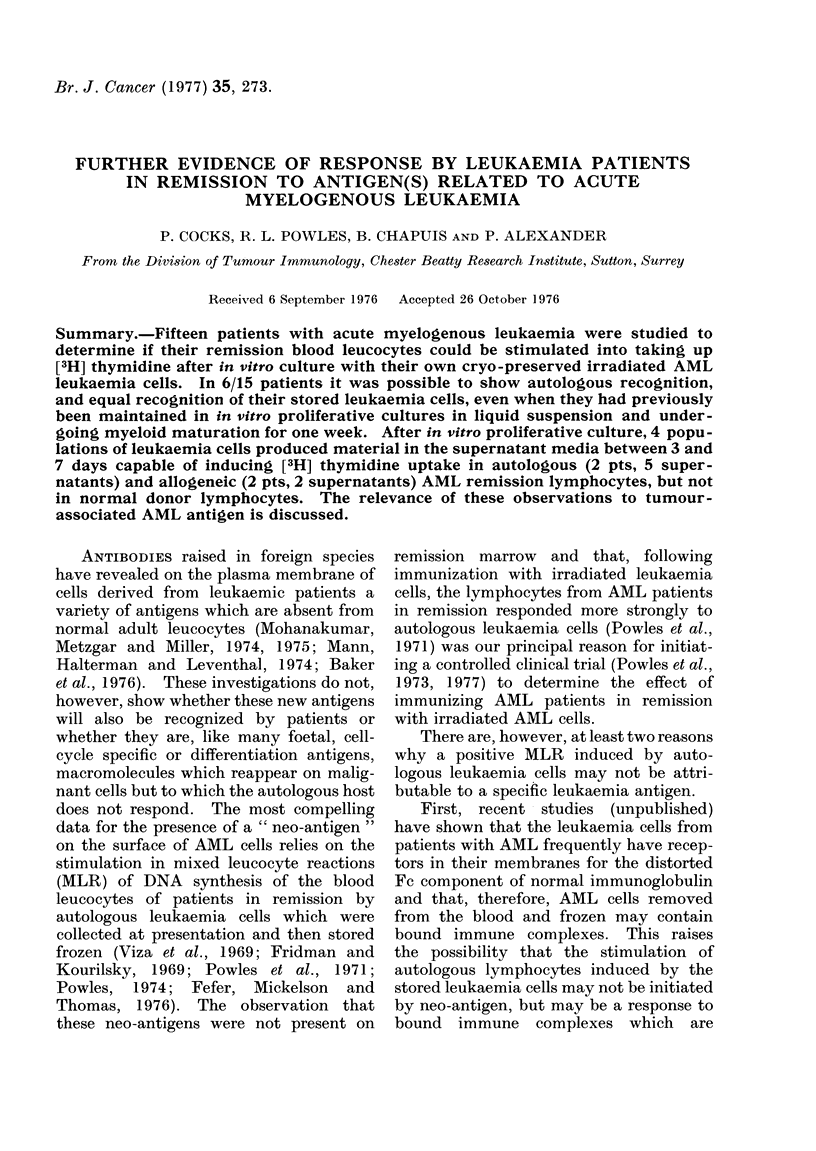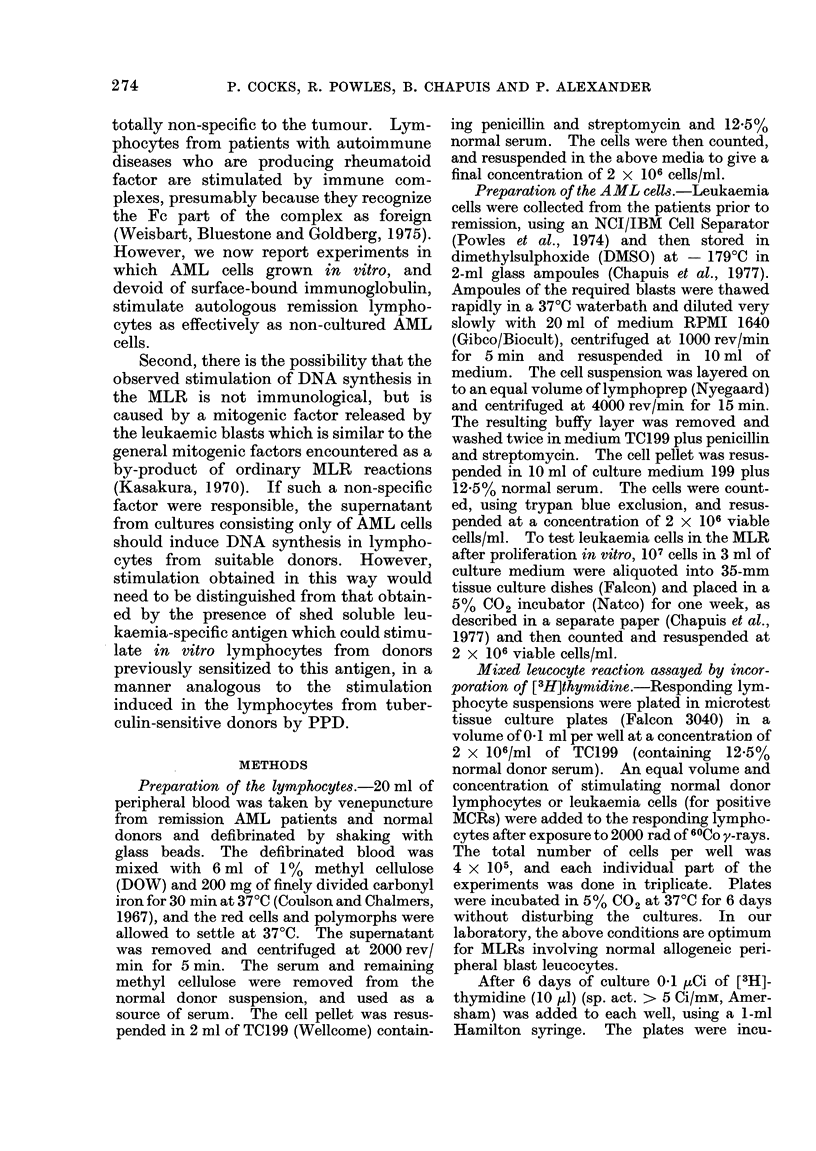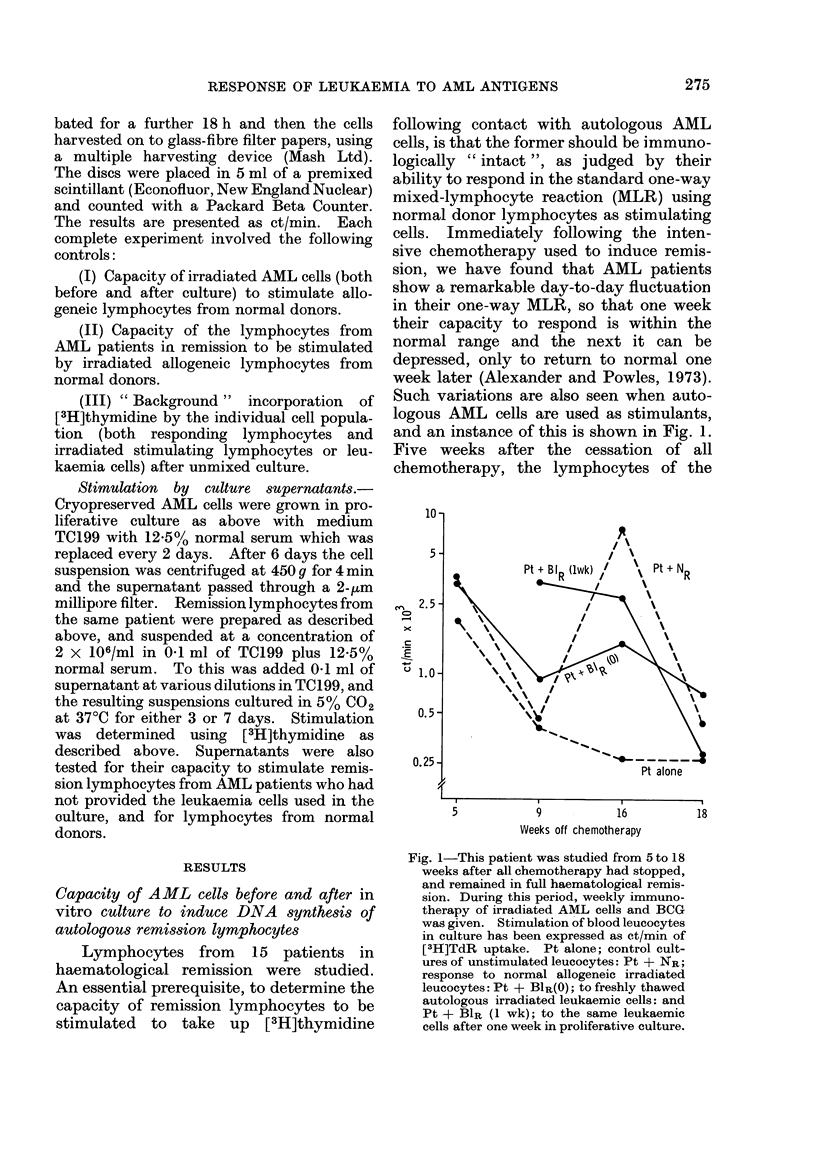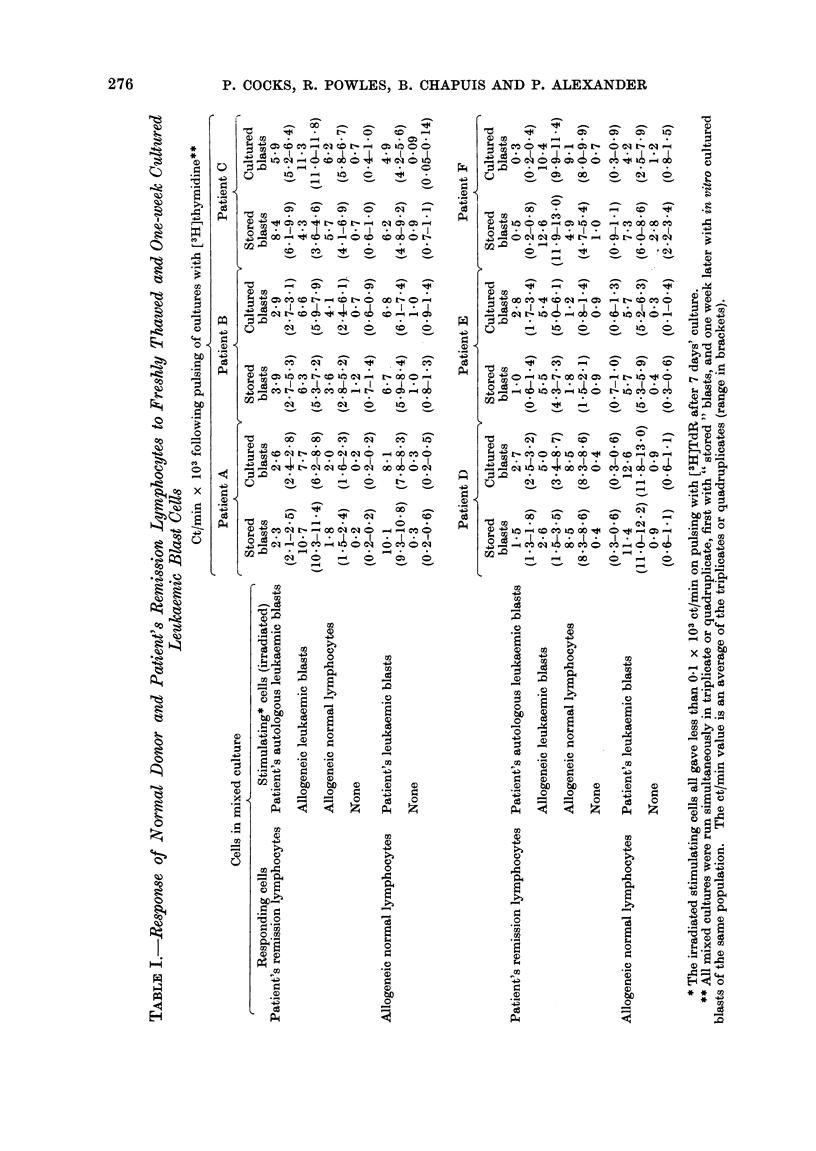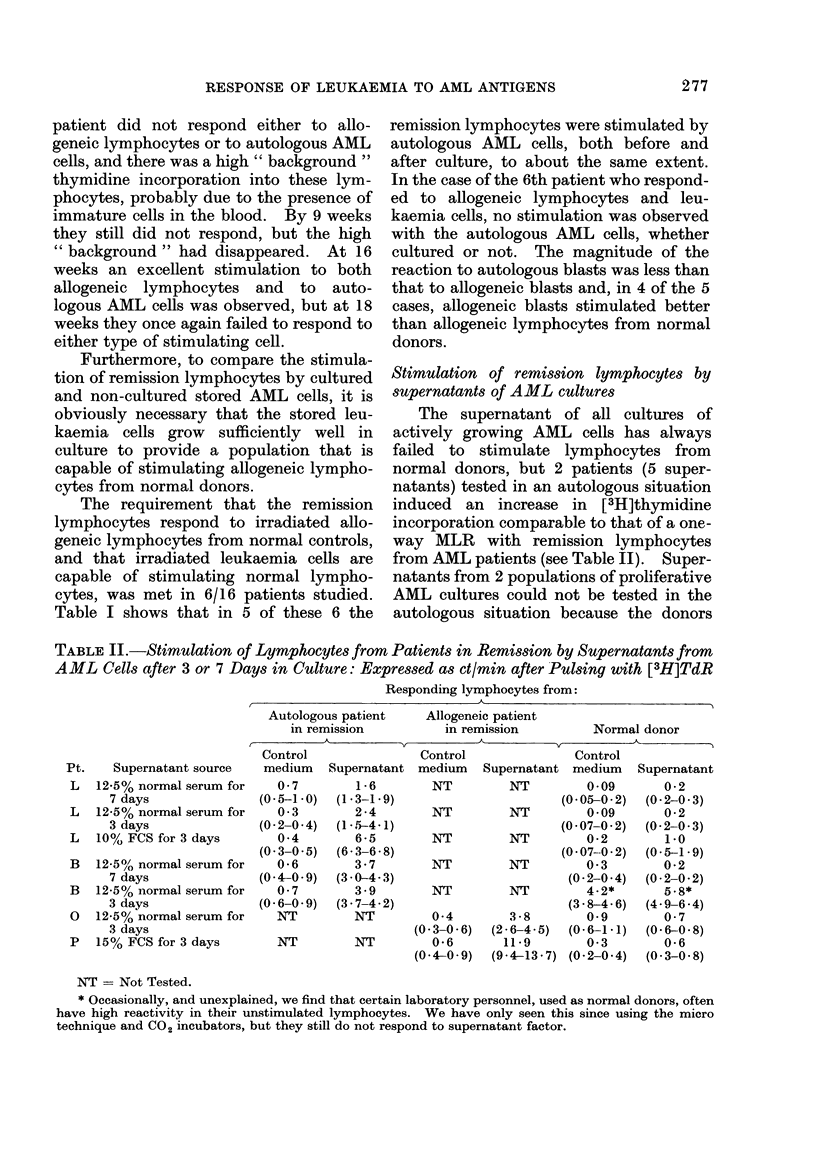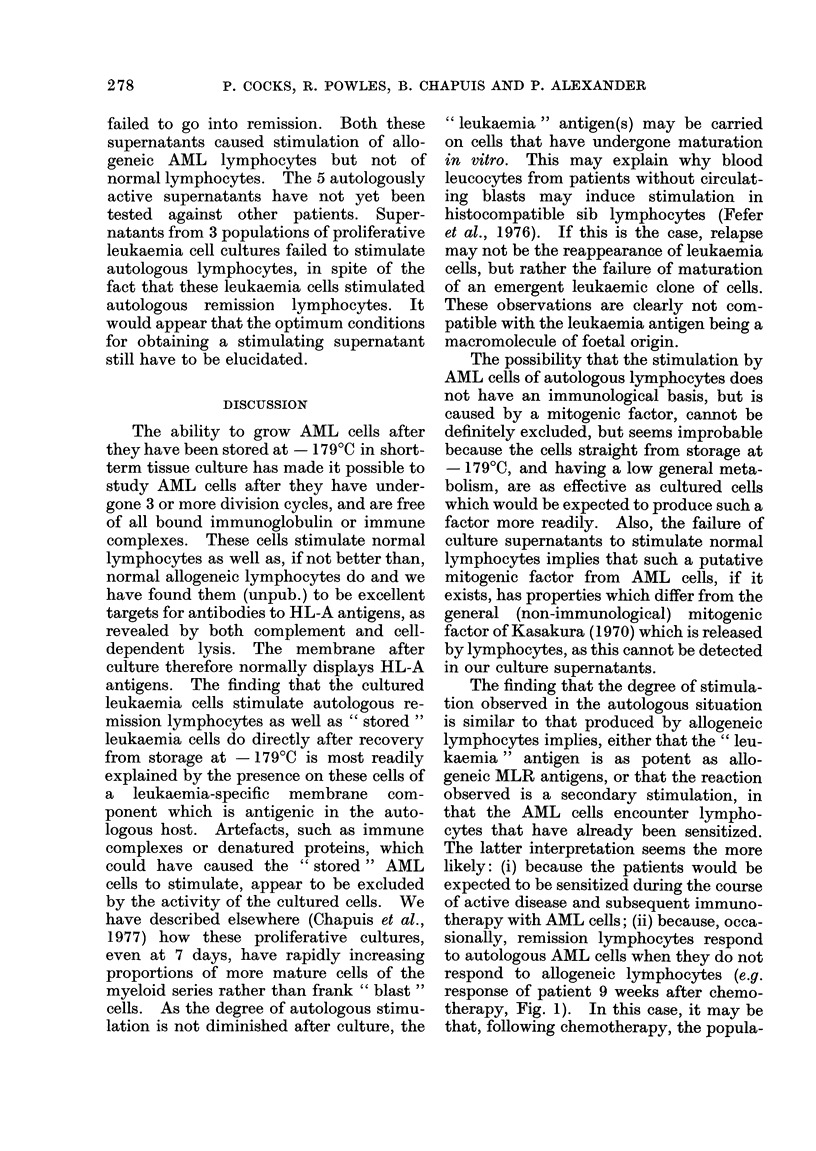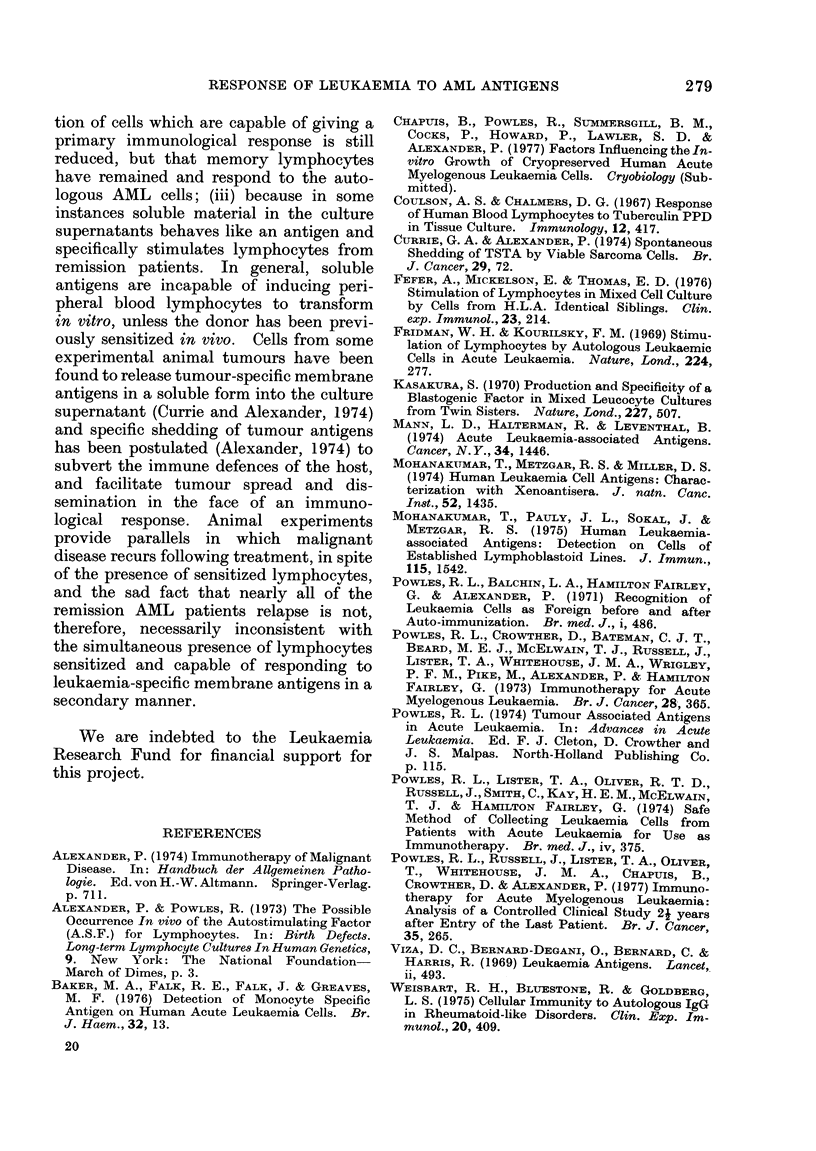# Further evidence of response by leukaemia patients in remission to antigen(s) related to acute myelogenous leukaemia.

**DOI:** 10.1038/bjc.1977.39

**Published:** 1977-03

**Authors:** P. Cocks, R. L. Powles, B. Chapuis, P. Alexander

## Abstract

Fifteen patients with acute myelogenous leukaemia were studied to determine if their remission blood leucocytes could be stimulated into taking up [3H] thymidine after in vitro culture with their own cryo-preserved irradiated AML leukaemia cells. In 6/15 patients it was possible to show autologous recognition and equal recognition of their stored leukaemia cells, even when they had previously been maintained in in vitro proliferative cultures in liquid suspension and undergoing myeloid maturation for one week. After in vitro proliferative culture, 4 populations of leukaemia cells produced material in the supernatant media between 3 and 7 days capable of inducing [3H] thymidine uptake in autologous (2 pts, 5 supernatants) and allogeneic (2 pts, 2 supernatants) AML remission lymphocytes, but not in normal donor lymphocytes. The relevance of these observations to tumour-associated AML antigen is discussed.


Br. J. Cancer (1977) 35, 273.

### FURTHER EVIDENCE OF RESPONSE BY LEUKAEMIA PATIENTS IN REMISSION TO ANTIGEN(S) RELATED TO ACUTE MYELOGENOUS LEUKAEMIA

P. COCKS, R. L. POWLES, B. CHAPUIS AND P. ALEXANDER

*From the Division of Tumour Immunology, Chester Beatty Research Institute, Sutton, Surrey*

Received 6 September 1976 Accepted 26 October 1976

**Summary.—Fifteen patients with acute myelogenous leukaemia were studied to determine if their remission blood leucocytes could be stimulated into taking up [$^3$H] thymidine after *in vitro* culture with their own cryo-preserved irradiated AML leukaemia cells. In 6/15 patients it was possible to show autologous recognition, and equal recognition of their stored leukaemia cells, even when they had previously been maintained in *in vitro* proliferative cultures in liquid suspension and undergoing myeloid maturation for one week. After *in vitro* proliferative culture, 4 populations of leukaemia cells produced material in the supernatant media between 3 and 7 days capable of inducing [$^3$H] thymidine uptake in autologous (2 pts, 5 supernatants) and allogeneic (2 pts, 2 supernatants) AML remission lymphocytes, but not in normal donor lymphocytes. The relevance of these observations to tumour-associated AML antigen is discussed.**

ANTIBODIES raised in foreign species have revealed on the plasma membrane of cells derived from leukaemic patients a variety of antigens which are absent from normal adult leucocytes (Mohanakumar, Metzgar and Miller, 1974, 1975; Mann, Halterman and Leventhal, 1974; Baker *et al.*, 1976). These investigations do not, however, show whether these new antigens will also be recognized by patients or whether they are, like many foetal, cell-cycle specific or differentiation antigens, macromolecules which reappear on malignant cells but to which the autologous host does not respond. The most compelling data for the presence of a " neo-antigen " on the surface of AML cells relies on the stimulation in mixed leucocyte reactions (MLR) of DNA synthesis of the blood leucocytes of patients in remission by autologous leukaemia cells which were collected at presentation and then stored frozen (Viza *et al.*, 1969; Fridman and Kourilsky, 1969; Powles *et al.*, 1971; Powles, 1974; Fefer, Mickelson and Thomas, 1976). The observation that these neo-antigens were not present on remission marrow and that, following immunization with irradiated leukaemia cells, the lymphocytes from AML patients in remission responded more strongly to autologous leukaemia cells (Powles *et al.*, 1971) was our principal reason for initiating a controlled clinical trial (Powles *et al.*, 1973, 1977) to determine the effect of immunizing AML patients in remission with irradiated AML cells.

There are, however, at least two reasons why a positive MLR induced by autologous leukaemia cells may not be attributable to a specific leukaemia antigen.

First, recent studies (unpublished) have shown that the leukaemia cells from patients with AML frequently have receptors in their membranes for the distorted Fc component of normal immunoglobulin and that, therefore, AML cells removed from the blood and frozen may contain bound immune complexes. This raises the possibility that the stimulation of autologous lymphocytes induced by the stored leukaemia cells may not be initiated by neo-antigen, but may be a response to bound immune complexes which are totally non-specific to the tumour. Lymphocytes from patients with autoimmune diseases who are producing rheumatoid factor are stimulated by immune complexes, presumably because they recognize the Fc part of the complex as foreign (Weisbart, Bluestone and Goldberg, 1975). However, we now report experiments in which AML cells grown *in vitro*, and devoid of surface-bound immunoglobulin, stimulate autologous remission lymphocytes as effectively as non-cultured AML cells.

Second, there is the possibility that the observed stimulation of DNA synthesis in the MLR is not immunological, but is caused by a mitogenic factor released by the leukaemic blasts which is similar to the general mitogenic factors encountered as a by-product of ordinary MLR reactions (Kasakura, 1970). If such a non-specific factor were responsible, the supernatant from cultures consisting only of AML cells should induce DNA synthesis in lymphocytes from suitable donors. However, stimulation obtained in this way would need to be distinguished from that obtained by the presence of shed soluble leukaemia-specific antigen which could stimulate *in vitro* lymphocytes from donors previously sensitized to this antigen, in a manner analogous to the stimulation induced in the lymphocytes from tuberculin-sensitive donors by PPD.

### METHODS

*Preparation of the lymphocytes.*—20 ml of peripheral blood was taken by venepuncture from remission AML patients and normal donors and defibrinated by shaking with glass beads. The defibrinated blood was mixed with 6 ml of 1% methyl cellulose (DOW) and 200 mg of finely divided carbonyl iron for 30 min at 37°C (Coulson and Chalmers, 1967), and the red cells and polymorphs were allowed to settle at 37°C. The supernatant was removed and centrifuged at 2000 rev/min for 5 min. The serum and remaining methyl cellulose were removed from the normal donor suspension, and used as a source of serum. The cell pellet was resuspended in 2 ml of TC199 (Wellcome) containing penicillin and streptomycin and 12·5% normal serum. The cells were then counted, and resuspended in the above media to give a final concentration of $2 \times 10^6$ cells/ml.

*Preparation of the AML cells.*—Leukaemia cells were collected from the patients prior to remission, using an NCI/IBM Cell Separator (Powles *et al.*, 1974) and then stored in dimethylsulphoxide (DMSO) at − 179°C in 2-ml glass ampoules (Chapuis *et al.*, 1977). Ampoules of the required blasts were thawed rapidly in a 37°C waterbath and diluted very slowly with 20 ml of medium RPMI 1640 (Gibco/Biocult), centrifuged at 1000 rev/min for 5 min and resuspended in 10 ml of medium. The cell suspension was layered on to an equal volume of lymphoprep (Nyegaard) and centrifuged at 4000 rev/min for 15 min. The resulting buffy layer was removed and washed twice in medium TC199 plus penicillin and streptomycin. The cell pellet was resuspended in 10 ml of culture medium 199 plus 12·5% normal serum. The cells were counted, using trypan blue exclusion, and resuspended at a concentration of $2 \times 10^6$ viable cells/ml. To test leukaemia cells in the MLR after proliferation *in vitro*, $10^7$ cells in 3 ml of culture medium were aliquoted into 35-mm tissue culture dishes (Falcon) and placed in a 5% $CO_2$ incubator (Natco) for one week, as described in a separate paper (Chapuis *et al.*, 1977) and then counted and resuspended at $2 \times 10^6$ viable cells/ml.

*Mixed leucocyte reaction assayed by incorporation of [$^3$H]thymidine.*—Responding lymphocyte suspensions were plated in microtest tissue culture plates (Falcon 3040) in a volume of 0·1 ml per well at a concentration of $2 \times 10^6$/ml of TC199 (containing 12·5% normal donor serum). An equal volume and concentration of stimulating normal donor lymphocytes or leukaemia cells (for positive MCRs) were added to the responding lymphocytes after exposure to 2000 rad of $^{60}$Co $\gamma$-rays. The total number of cells per well was $4 \times 10^5$, and each individual part of the experiments was done in triplicate. Plates were incubated in 5% $CO_2$ at 37°C for 6 days without disturbing the cultures. In our laboratory, the above conditions are optimum for MLRs involving normal allogeneic peripheral blast leucocytes.

After 6 days of culture 0·1 $\mu$Ci of [$^3$H]-thymidine (10 $\mu$l) (sp. act. > 5 Ci/mM, Amersham) was added to each well, using a 1-ml Hamilton syringe. The plates were incubated for a further 18 h and then the cells harvested on to glass-fibre filter papers, using a multiple harvesting device (Mash Ltd). The discs were placed in 5 ml of a premixed scintillant (Econofluor, New England Nuclear) and counted with a Packard Beta Counter. The results are presented as ct/min. Each complete experiment involved the following controls:

(I) Capacity of irradiated AML cells (both before and after culture) to stimulate allogeneic lymphocytes from normal donors.

(II) Capacity of the lymphocytes from AML patients in remission to be stimulated by irradiated allogeneic lymphocytes from normal donors.

(III) " Background " incorporation of [$^3$H]thymidine by the individual cell population (both responding lymphocytes and irradiated stimulating lymphocytes or leukaemia cells) after unmixed culture.

*Stimulation by culture supernatants.*—Cryopreserved AML cells were grown in proliferative culture as above with medium TC199 with 12·5% normal serum which was replaced every 2 days. After 6 days the cell suspension was centrifuged at 450 *g* for 4 min and the supernatant passed through a 2-$\mu$m millipore filter. Remission lymphocytes from the same patient were prepared as described above, and suspended at a concentration of $2 \times 10^6$/ml in 0·1 ml of TC199 plus 12·5% normal serum. To this was added 0·1 ml of supernatant at various dilutions in TC199, and the resulting suspensions cultured in 5% $CO_2$ at 37°C for either 3 or 7 days. Stimulation was determined using [$^3$H]thymidine as described above. Supernatants were also tested for their capacity to stimulate remission lymphocytes from AML patients who had not provided the leukaemia cells used in the culture, and for lymphocytes from normal donors.

### RESULTS

### *Capacity of AML cells before and after* in vitro *culture to induce DNA synthesis of autologous remission lymphocytes*

Lymphocytes from 15 patients in haematological remission were studied. An essential prerequisite, to determine the capacity of remission lymphocytes to be stimulated to take up [$^3$H]thymidine following contact with autologous AML cells, is that the former should be immunologically " intact ", as judged by their ability to respond in the standard one-way mixed-lymphocyte reaction (MLR) using normal donor lymphocytes as stimulating cells. Immediately following the intensive chemotherapy used to induce remission, we have found that AML patients show a remarkable day-to-day fluctuation in their one-way MLR, so that one week their capacity to respond is within the normal range and the next it can be depressed, only to return to normal one week later (Alexander and Powles, 1973). Such variations are also seen when autologous AML cells are used as stimulants, and an instance of this is shown in Fig. 1. Five weeks after the cessation of all chemotherapy, the lymphocytes of the

Fig. 1—This patient was studied from 5 to 18 weeks after all chemotherapy had stopped, and remained in full haematological remission. During this period, weekly immunotherapy of irradiated AML cells and BCG was given. Stimulation of blood leucocytes in culture has been expressed as ct/min of [$^3$H]TdR uptake. Pt alone; control cultures of unstimulated leucocytes: Pt + $N_R$; response to normal allogeneic irradiated leucocytes: Pt + $Bl_R(0)$; to freshly thawed autologous irradiated leukaemic cells: and Pt + $Bl_R$ (1 wk); to the same leukaemic cells after one week in proliferative culture.

TABLE I.—*Response of Normal Donor and Patient's Remission Lymphocytes to Freshly Thawed and One-week Cultured Leukaemic Blast Cells*

Ct/min × $10^3$ following pulsing of cultures with [$^3$H]thymidine**

| Responding cells | Stimulating* cells (irradiated) | Patient A Stored blasts | Patient A Cultured blasts | Patient B Stored blasts | Patient B Cultured blasts | Patient C Stored blasts | Patient C Cultured blasts |
|---|---|---|---|---|---|---|---|
| Patient's remission lymphocytes | Patient's autologous leukaemic blasts | 2·3 (2·1–2·5) | 2·6 (2·4–2·8) | 3·9 (2·7–5·3) | 2·9 (2·7–3·1) | 8·4 (6·1–9·9) | 5·9 (5·2–6·4) |
| | Allogeneic leukaemic blasts | 10·7 (10·3–11·4) | 7·7 (6·2–8·8) | 6·3 (5·3–7·2) | 6·6 (5·9–7·9) | 4·3 (3·6–4·6) | 11·3 (11·0–11·8) |
| | Allogeneic normal lymphocytes | 1·8 (1·5–2·4) | 2·0 (1·6–2·3) | 3·6 (2·8–5·2) | 4·1 (2·4–6·1) | 5·7 (4·1–6·9) | 6·2 (5·8–6·7) |
| | None | 0·2 (0·2–0·2) | 0·2 (0·2–0·2) | 1·2 (0·7–1·4) | 0·7 (0·6–0·9) | 0·7 (0·6–1·0) | 0·7 (0·4–1·0) |
| Allogeneic normal lymphocytes | Patient's leukaemic blasts | 10·1 (9·3–10·8) | 8·1 (7·8–8·3) | 6·7 (5·9–8·4) | 6·8 (6·1–7·4) | 6·2 (4·8–9·2) | 4·9 (4·2–5·6) |
| | None | 0·3 (0·2–0·6) | 0·3 (0·2–0·5) | 1·0 (0·8–1·3) | 1·0 (0·9–1·4) | 0·9 (0·7–1·1) | 0·09 (0·05–0·14) |

| Responding cells | Stimulating* cells (irradiated) | Patient D Stored blasts | Patient D Cultured blasts | Patient E Stored blasts | Patient E Cultured blasts | Patient F Stored blasts | Patient F Cultured blasts |
|---|---|---|---|---|---|---|---|
| Patient's remission lymphocytes | Patient's autologous leukaemic blasts | 1·5 (1·3–1·8) | 2·7 (2·5–3·2) | 1·0 (0·6–1·4) | 2·8 (1·7–3·4) | 0·5 (0·2–0·8) | 0·3 (0·2–0·4) |
| | Allogeneic leukaemic blasts | 2·6 (1·5–3·5) | 5·0 (3·4–8·7) | 5·5 (4·3–7·3) | 5·4 (5·0–6·1) | 12·6 (11·9–13·0) | 10·4 (9·9–11·4) |
| | Allogeneic normal lymphocytes | 8·5 (8·3–8·6) | 8·5 (8·3–8·6) | 1·8 (1·5–2·1) | 1·2 (0·8–1·4) | 4·9 (4·7–5·4) | 9·1 (8·0–9·9) |
| | None | 0·4 (0·3–0·6) | 0·4 (0·3–0·6) | 0·9 (0·7–1·0) | 0·9 (0·6–1·3) | 1·0 (0·9–1·1) | 0·7 (0·3–0·9) |
| Allogeneic normal lymphocytes | Patient's leukaemic blasts | 11·4 (11·0–12·2) | 12·6 (11·8–13·0) | 5·7 (5·3–5·9) | 5·7 (5·2–6·3) | 7·3 (6·0–8·6) | 4·2 (2·5–7·9) |
| | None | 0·9 (0·6–1·1) | 0·9 (0·6–1·1) | 0·4 (0·3–0·6) | 0·3 (0·1–0·4) | 2·8 (2·2–3·4) | 1·2 (0·8–1·5) |

* The irradiated stimulating cells all gave less than 0·1 × $10^3$ ct/min on pulsing with [$^3$H]TdR after 7 days' culture.

** All mixed cultures were run simultaneously in triplicate or quadruplicate, first with " stored " blasts, and one week later with *in vitro* cultured blasts of the same population. The ct/min value is an average of the triplicates or quadruplicates (range in brackets).

patient did not respond either to allogeneic lymphocytes or to autologous AML cells, and there was a high " background " thymidine incorporation into these lymphocytes, probably due to the presence of immature cells in the blood. By 9 weeks they still did not respond, but the high " background " had disappeared. At 16 weeks an excellent stimulation to both allogeneic lymphocytes and to autologous AML cells was observed, but at 18 weeks they once again failed to respond to either type of stimulating cell.

Furthermore, to compare the stimulation of remission lymphocytes by cultured and non-cultured stored AML cells, it is obviously necessary that the stored leukaemia cells grow sufficiently well in culture to provide a population that is capable of stimulating allogeneic lymphocytes from normal donors.

The requirement that the remission lymphocytes respond to irradiated allogeneic lymphocytes from normal controls, and that irradiated leukaemia cells are capable of stimulating normal lymphocytes, was met in 6/16 patients studied. Table I shows that in 5 of these 6 the remission lymphocytes were stimulated by autologous AML cells, both before and after culture, to about the same extent. In the case of the 6th patient who responded to allogeneic lymphocytes and leukaemia cells, no stimulation was observed with the autologous AML cells, whether cultured or not. The magnitude of the reaction to autologous blasts was less than that to allogeneic blasts and, in 4 of the 5 cases, allogeneic blasts stimulated better than allogeneic lymphocytes from normal donors.

*Stimulation of remission lymphocytes by supernatants of AML cultures*

The supernatant of all cultures of actively growing AML cells has always failed to stimulate lymphocytes from normal donors, but 2 patients (5 supernatants) tested in an autologous situation induced an increase in [$^3$H]thymidine incorporation comparable to that of a one-way MLR with remission lymphocytes from AML patients (see Table II). Supernatants from 2 populations of proliferative AML cultures could not be tested in the autologous situation because the donors

TABLE II.—*Stimulation of Lymphocytes from Patients in Remission by Supernatants from AML Cells after 3 or 7 Days in Culture: Expressed as ct/min after Pulsing with [$^3H$]TdR*

| | | Responding lymphocytes from: | | | | | |
|---|---|---|---|---|---|---|---|
| | | Autologous patient in remission | | Allogeneic patient in remission | | Normal donor | |
| Pt. | Supernatant source | Control medium | Supernatant | Control medium | Supernatant | Control medium | Supernatant |
| L | 12·5% normal serum for 7 days | 0·7 (0·5–1·0) | 1·6 (1·3–1·9) | NT | NT | 0·09 (0·05–0·2) | 0·2 (0·2–0·3) |
| L | 12·5% normal serum for 3 days | 0·3 (0·2–0·4) | 2·4 (1·5–4·1) | NT | NT | 0·09 (0·07–0·2) | 0·2 (0·2–0·3) |
| L | 10% FCS for 3 days | 0·4 (0·3–0·5) | 6·5 (6·3–6·8) | NT | NT | 0·2 (0·07–0·2) | 1·0 (0·5–1·9) |
| B | 12·5% normal serum for 7 days | 0·6 (0·4–0·9) | 3·7 (3·0–4·3) | NT | NT | 0·3 (0·2–0·4) | 0·2 (0·2–0·2) |
| B | 12·5% normal serum for 3 days | 0·7 (0·6–0·9) | 3·9 (3·7–4·2) | NT | NT | 4·2* (3·8–4·6) | 5·8* (4·9–6·4) |
| O | 12·5% normal serum for 3 days | NT | NT | 0·4 (0·3–0·6) | 3·8 (2·6–4·5) | 0·9 (0·6–1·1) | 0·7 (0·6–0·8) |
| P | 15% FCS for 3 days | NT | NT | 0·6 (0·4–0·9) | 11·9 (9·4–13·7) | 0·3 (0·2–0·4) | 0·6 (0·3–0·8) |

NT = Not Tested.

* Occasionally, and unexplained, we find that certain laboratory personnel, used as normal donors, often have high reactivity in their unstimulated lymphocytes. We have only seen this since using the micro technique and $CO_2$ incubators, but they still do not respond to supernatant factor.

failed to go into remission. Both these supernatants caused stimulation of allogeneic AML lymphocytes but not of normal lymphocytes. The 5 autologously active supernatants have not yet been tested against other patients. Supernatants from 3 populations of proliferative leukaemia cell cultures failed to stimulate autologous lymphocytes, in spite of the fact that these leukaemia cells stimulated autologous remission lymphocytes. It would appear that the optimum conditions for obtaining a stimulating supernatant still have to be elucidated.

### DISCUSSION

The ability to grow AML cells after they have been stored at — 179°C in short-term tissue culture has made it possible to study AML cells after they have undergone 3 or more division cycles, and are free of all bound immunoglobulin or immune complexes. These cells stimulate normal lymphocytes as well as, if not better than, normal allogeneic lymphocytes do and we have found them (unpub.) to be excellent targets for antibodies to HL-A antigens, as revealed by both complement and cell-dependent lysis. The membrane after culture therefore normally displays HL-A antigens. The finding that the cultured leukaemia cells stimulate autologous remission lymphocytes as well as " stored " leukaemia cells do directly after recovery from storage at — 179°C is most readily explained by the presence on these cells of a leukaemia-specific membrane component which is antigenic in the autologous host. Artefacts, such as immune complexes or denatured proteins, which could have caused the " stored " AML cells to stimulate, appear to be excluded by the activity of the cultured cells. We have described elsewhere (Chapuis *et al.*, 1977) how these proliferative cultures, even at 7 days, have rapidly increasing proportions of more mature cells of the myeloid series rather than frank " blast " cells. As the degree of autologous stimulation is not diminished after culture, the " leukaemia " antigen(s) may be carried on cells that have undergone maturation *in vitro*. This may explain why blood leucocytes from patients without circulating blasts may induce stimulation in histocompatible sib lymphocytes (Fefer *et al.*, 1976). If this is the case, relapse may not be the reappearance of leukaemia cells, but rather the failure of maturation of an emergent leukaemic clone of cells. These observations are clearly not compatible with the leukaemia antigen being a macromolecule of foetal origin.

The possibility that the stimulation by AML cells of autologous lymphocytes does not have an immunological basis, but is caused by a mitogenic factor, cannot be definitely excluded, but seems improbable because the cells straight from storage at — 179°C, and having a low general metabolism, are as effective as cultured cells which would be expected to produce such a factor more readily. Also, the failure of culture supernatants to stimulate normal lymphocytes implies that such a putative mitogenic factor from AML cells, if it exists, has properties which differ from the general (non-immunological) mitogenic factor of Kasakura (1970) which is released by lymphocytes, as this cannot be detected in our culture supernatants.

The finding that the degree of stimulation observed in the autologous situation is similar to that produced by allogeneic lymphocytes implies, either that the " leukaemia " antigen is as potent as allogeneic MLR antigens, or that the reaction observed is a secondary stimulation, in that the AML cells encounter lymphocytes that have already been sensitized. The latter interpretation seems the more likely: (i) because the patients would be expected to be sensitized during the course of active disease and subsequent immunotherapy with AML cells; (ii) because, occasionally, remission lymphocytes respond to autologous AML cells when they do not respond to allogeneic lymphocytes (*e.g.* response of patient 9 weeks after chemotherapy, Fig. 1). In this case, it may be that, following chemotherapy, the population of cells which are capable of giving a primary immunological response is still reduced, but that memory lymphocytes have remained and respond to the autologous AML cells; (iii) because in some instances soluble material in the culture supernatants behaves like an antigen and specifically stimulates lymphocytes from remission patients. In general, soluble antigens are incapable of inducing peripheral blood lymphocytes to transform *in vitro*, unless the donor has been previously sensitized *in vivo*. Cells from some experimental animal tumours have been found to release tumour-specific membrane antigens in a soluble form into the culture supernatant (Currie and Alexander, 1974) and specific shedding of tumour antigens has been postulated (Alexander, 1974) to subvert the immune defences of the host, and facilitate tumour spread and dissemination in the face of an immunological response. Animal experiments provide parallels in which malignant disease recurs following treatment, in spite of the presence of sensitized lymphocytes, and the sad fact that nearly all of the remission AML patients relapse is not, therefore, necessarily inconsistent with the simultaneous presence of lymphocytes sensitized and capable of responding to leukaemia-specific membrane antigens in a secondary manner.

We are indebted to the Leukaemia Research Fund for financial support for this project.

### REFERENCES

ALEXANDER, P. (1974) Immunotherapy of Malignant Disease. In: *Handbuch der Allgemeinen Pathologie.* Ed. von H.-W. Altmann. Springer-Verlag. p. 711.

ALEXANDER, P. & POWLES, R. (1973) The Possible Occurrence *In vivo* of the Autostimulating Factor (A.S.F.) for Lymphocytes. In: *Birth Defects. Long-term Lymphocyte Cultures In Human Genetics*, **9**. New York: The National Foundation—March of Dimes, p. 3.

BAKER, M. A., FALK, R. E., FALK, J. & GREAVES, M. F. (1976) Detection of Monocyte Specific Antigen on Human Acute Leukaemia Cells. *Br. J. Haem.*, **32**, 13.

CHAPUIS, B., POWLES, R., SUMMERSGILL, B. M., COCKS, P., HOWARD, P., LAWLER, S. D. & ALEXANDER, P. (1977) Factors Influencing the *In-vitro* Growth of Cryopreserved Human Acute Myelogenous Leukaemia Cells. *Cryobiology* (Submitted).

COULSON, A. S. & CHALMERS, D. G. (1967) Response of Human Blood Lymphocytes to Tuberculin PPD in Tissue Culture. *Immunology*, **12**, 417.

CURRIE, G. A. & ALEXANDER, P. (1974) Spontaneous Shedding of TSTA by Viable Sarcoma Cells. *Br. J. Cancer*, **29**, 72.

FEFER, A., MICKELSON, E. & THOMAS, E. D. (1976) Stimulation of Lymphocytes in Mixed Cell Culture by Cells from H.L.A. Identical Siblings. *Clin. exp. Immunol.*, **23**, 214.

FRIDMAN, W. H. & KOURILSKY, F. M. (1969) Stimulation of Lymphocytes by Autologous Leukaemic Cells in Acute Leukaemia. *Nature, Lond.*, **224**, 277.

KASAKURA, S. (1970) Production and Specificity of a Blastogenic Factor in Mixed Leucocyte Cultures from Twin Sisters. *Nature, Lond.*, **227**, 507.

MANN, L. D., HALTERMAN, R. & LEVENTHAL, B. (1974) Acute Leukaemia-associated Antigens. *Cancer, N.Y.*, **34**, 1446.

MOHANAKUMAR, T., METZGAR, R. S. & MILLER, D. S. (1974) Human Leukaemia Cell Antigens: Characterization with Xenoantisera. *J. natn. Canc. Inst.*, **52**, 1435.

MOHANAKUMAR, T., PAULY, J. L., SOKAL, J. & METZGAR, R. S. (1975) Human Leukaemia-associated Antigens: Detection on Cells of Established Lymphoblastoid Lines. *J. Immun.*, **115**, 1542.

POWLES, R. L., BALCHIN, L. A., HAMILTON FAIRLEY, G. & ALEXANDER, P. (1971) Recognition of Leukaemia Cells as Foreign before and after Auto-immunization. *Br. med. J.*, i, 486.

POWLES, R. L., CROWTHER, D., BATEMAN, C. J. T., BEARD, M. E. J., MCELWAIN, T. J., RUSSELL, J., LISTER, T. A., WHITEHOUSE, J. M. A., WRIGLEY, P. F. M., PIKE, M., ALEXANDER, P. & HAMILTON FAIRLEY, G. (1973) Immunotherapy for Acute Myelogenous Leukaemia. *Br. J. Cancer*, **28**, 365.

POWLES, R. L. (1974) Tumour Associated Antigens in Acute Leukaemia. In: *Advances in Acute Leukaemia.* Ed. F. J. Cleton, D. Crowther and J. S. Malpas. North-Holland Publishing Co. p. 115.

POWLES, R. L., LISTER, T. A., OLIVER, R. T. D., RUSSELL, J., SMITH, C., KAY, H. E. M., MCELWAIN, T. J. & HAMILTON FAIRLEY, G. (1974) Safe Method of Collecting Leukaemia Cells from Patients with Acute Leukaemia for Use as Immunotherapy. *Br. med. J.*, iv, 375.

POWLES, R. L., RUSSELL, J., LISTER, T. A., OLIVER, T., WHITEHOUSE, J. M. A., CHAPUIS, B., CROWTHER, D. & ALEXANDER, P. (1977) Immunotherapy for Acute Myelogenous Leukaemia: Analysis of a Controlled Clinical Study 2½ years after Entry of the Last Patient. *Br. J. Cancer*, **35**, 265.

VIZA, D. C., BERNARD-DEGANI, O., BERNARD, C. & HARRIS, R. (1969) Leukaemia Antigens. *Lancet*, ii, 493.

WEISBART, R. H., BLUESTONE, R. & GOLDBERG, L. S. (1975) Cellular Immunity to Autologous IgG in Rheumatoid-like Disorders. *Clin. Exp. Immunol.*, **20**, 409.